# On Albuminuria, or Bright’s Disease

**Published:** 1844-06-01

**Authors:** 


### ON ALBUMINURIA, OR BRIGHT'S DISEASE.

The following case, which occurred in the wards of M. Piorry, furnished this distinguished professor an opportunity of pointing out to the students who attend his clinical lectures, how useful plessimetry is in establishing the diagnosis. M. ——, ætat 45, strong constitution, suffering for a twelvemonth past from Bright's disease, for which he had already been treated in several hospitals. On the 25th February, 1844, he was received in the wards of Professor Piorry, at the *Pitié*, and presented the following symptoms:—The pain which existed in the lumbar region has, the last two days, increased considerably; the quantity of urine passed in the twenty-four hours is not more than lbiss.; it is pale, thick, of a disagreeable smell, and forms, on cooling, a filamentous, flaky deposit. When nitric acid is added, an abundant white sediment is precipitated; and when heated in a glass-tube, the coagulum formed is considerable. Fever; pulse 120, strong; no anasarca. The results obtained, on examining by percussion the kidneys, differed;—thus, on the right side, the organ is of the natural size; whereas, on the left, it had considerably increased. The diagnosis thus established, the patient was ordered to drink freely about fourteen to sixteen pints of tisanne daily; but this treatment, instead of alleviating the sufferings of the patient, on the contrary aggravated them; and what is still more remarkable, by percussion, the right kidney was found to have become twice the size it was when previously examined. This effect, caused by liquids drunk in large quantities, has as yet never been recorded. The next day all liquids were forbidden, the patient was put on a *dry regimen*, and from this moment the quantity of urine decreased, and, two days after, the kidney had diminished to its primitive size. The albumine, which had increased in quantity, as soon as the patient drank so much, on the contrary began to decrease when he was prevented taking any liquid—so much so, that in eight days, nitric acid and heat showed that the urine contained a very small quantity. The fever had disappeared, and the patient appeared in a very fair way of recovery. His diet was composed exclusively of roast meat, and in order to quench his thirst, he was ordered to take a few drops of water in his mouth, and then spit them out. Ten days after his entry, he complained of smart pain in the right fossa iliaca, which seemed to follow the direction of the ureter, for which twenty leeches were applied, and afterwards emollient poultices. The patient remained in this state a fortnight longer, when all at once he was taken with violent pain in the præcordia, accompanied with intense dyspnœa and fever. By percussion the dulness in this region (about six and a half inches transversely, and nearly six vertically) was found to be considerable; the hand placed on this part did not feel any tremor. Auscultation indicated the presence of the friction sound, masking completely the first sound of the heart, and continuing nearly the whole of the first interval; the harsh sound offers its maximum of intensity over the right ventricle, near the left border of the sternum. Nothing abnormal in the large vessels. Evidently the patient was now affected with pericarditis, and notwithstanding an active anti-phlogistic treatment, he died five days after.

*Post Mortem Examination.*—The two kidneys had had increased in size; the adipose capsules which envelope them were rather increased in size, and adherent. When cut into, the cortical substance was found to be pale, of a yellowish tint, with slight traces of injection; no granulations. Each kidney presented, at its upper extremity, a cyst filled with serosity, of the size of a small walnut, which had developed itself in the cortical substance: several others, of the size of a pea, were disseminated here and there. The tubular substance healthy. In the right fossa iliaca, in the spot where the patient experienced such pain there existed under the aponeurosis, and in the substance of the iliacus internus muscle, an abscess, which offered no connection whatever with the disease of the kidneys, and whose cause was unknown. The infundibula, the pelvis of the ureters, and the ureters themselves, healthy. Finally, on examining the heart, all the characteristic signs of pericarditis were discovered.—While on this subject, we may notice an article published in the last number of the *Journal des Connaissances Medico-Chirurgicales*, in which the writer, after enumerating the different authors who have studied this disease, from the days of Hippocrates down to the present time, classes the different varieties under the following heads:—1°. Albuminous nephritis perfectly characterized.—2°. Albuminuria without nephritis, but complicated with dropsy.—3°. Albuminuria without nephritis or dropsy.—And concludes—that Albuminuria is a humoral disease, produced by a peculiar alteration of the blood and of the renal secretion,—that it is not absolutely necessary that the kidney should be altered in its texture to give rise to this disease,—that the blood is generally changed in its composition, let the origin of the disease be what it may,—that when the albuminuria is not caused by nephritis, it is owing to disorders which act on the whole system, such as cancers of the uterus and stomach, typhoid fevers, phthisis, scarlatina, chlorosis, &c.,—that it may be asked whether, in albuminous nephritis, albuminuria does not precede the lesion of the kidney, especially when this takes place after scarlatina,—that the divisions here established may become important in a therapeutical point of view, and albuminuria possess special remedies, as chlorosis, &c. In the meanwhile its history, though still incomplete, is an important addition to that of humorism, renovated and enlightened by chemistry and pathological anatomy. The discovery made by Bright is one of the most useful of the present day, and is sufficient to immortalize his name.—*London Medical Times.*

